# Visualization of the High Surface-to-Volume Ratio of Nanomaterials and Its Consequences

**DOI:** 10.1021/acs.jchemed.4c00089

**Published:** 2024-07-03

**Authors:** Maria Pozzi, Sarodi Jonak Dutta, Mia Kuntze, Jeannette Bading, Johanna S. Rüßbült, Cornelius Fabig, Malte Langfeldt, Florian Schulz, Patricia Horcajada, Wolfgang J. Parak

**Affiliations:** † Fachbereich Physik, Universität Hamburg, 22607 Hamburg, Germany; ‡ Ratsgymnasium Rotenburg, 27356 Rotenburg, Germany; § Advanced Porous Materials Unit, IMDEA Energy Institute, 28935 Móstoles, Madrid, Spain

**Keywords:** Surface-to-Volume Ratio, Nanoparticles, Surface Reactivity, Downsizing of Materials

## Abstract

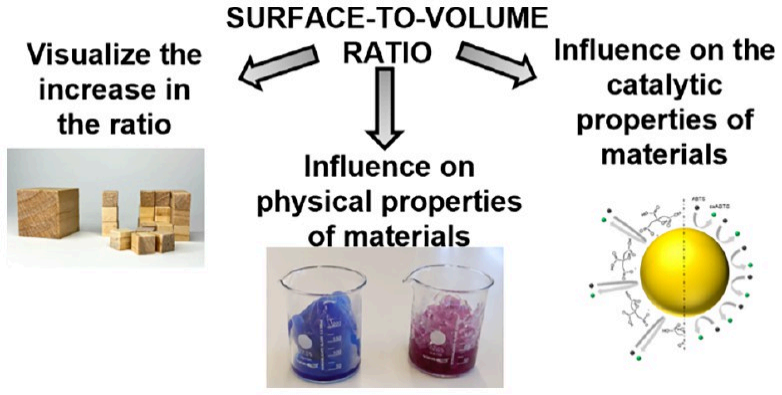

When bulk materials are reduced in size to the nanometer scale, in particular, their surface-to-volume ratio increases drastically. We introduce some simple experiments on how to visualize this concept to students in the framework of a laboratory class. In the same context, experiments to demonstrate the consequences of this on the properties of the materials are introduced. This will involve solubility and chemical surface reactivity of the materials and properties originated from the surface. In the framework of their chemical reactivity, potential benefits and threads of nanomaterials due to their high surface-to-volume ratio will be discussed, such as applications as catalysts and their impact on nanotoxicology.

## Introduction: How to Visualize the Increase in Surface-to-Volume Ratio

The high surface-to-volume ratio of nanomaterials is one of their striking properties. For a student, it is important to understand that, when going from bulk to nanomaterials, the material itself remains the same; i.e., there is no chemical reaction, no change of atoms, but there is the introduction of a new surface.^1−3^ The easiest way for visualizing the concept of high surface-to-volume ratio is to assemble and disassemble a large cube into many small cubes. For this, we built 3 × 3 × 3 = 27 “small” cubes of 1 cm side length and one “big” cube of 3 cm side length and showed how the “small” cubes can be assembled into a “big” cube and again be disassembled to “small” cubes. While this obviously is a simple experiment, we can perform it in several variants to demonstrate different aspects.

### Variant (i): The Use of Wooden Cubes, Allowing for Direct Comparison of Volumes (Masses)

**Figure 1:**
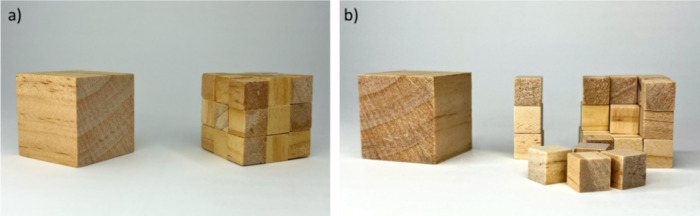
(a) 27 “small” wooden cubes are assembled to form a “big” cube (right), having the same volume as the “big” wooden cube shown on the left. (b) The “big” cube on the right is disassembled to 27 “small” cubes. While the total volume is retained, the total surface area increases.

The teacher needs a wooden cube of 3 cm in length and 27 wooden cubes of 1 cm side length. If the teacher has no possibility to get such cubes made from a machine shop, they can also be made using dice instead of wooden cubes. Twenty-seven dice are glued together to form a big dice (cf., Video V1), and the other 27 dice remain as they are. For the wooden cubes, 27 “small” cubes are assembled together to form a “big” cube and are put next to the “big” cube to show that both the assembly of “small” cubes and the “big” cube have the same volume (Figure 1, Video V2). In order to further demonstrate this, first the 27 “small” cubes and then the “big” cube are weighted with a balance (Video V3). As the material (wood) and, thus, the density are the same, the mass reflects the volume of the cubes.^4^ It would be most likely that the “small” cubes and the “big” cube will not weigh exactly the same as the last digit of the balance; the teacher can give a short discourse about experimental errors. This first experiment should show the students that a “big” cube can be emulated by many “small” cubes. Or, in other words, a “big” piece of material contains many “small” pieces of the same material. In the next step, the students should now understand the order of magnitude of this concept.

### Variant (ii): The Use of Paper Cubes, Allowing Students for Manual Work

**Figure 2:**
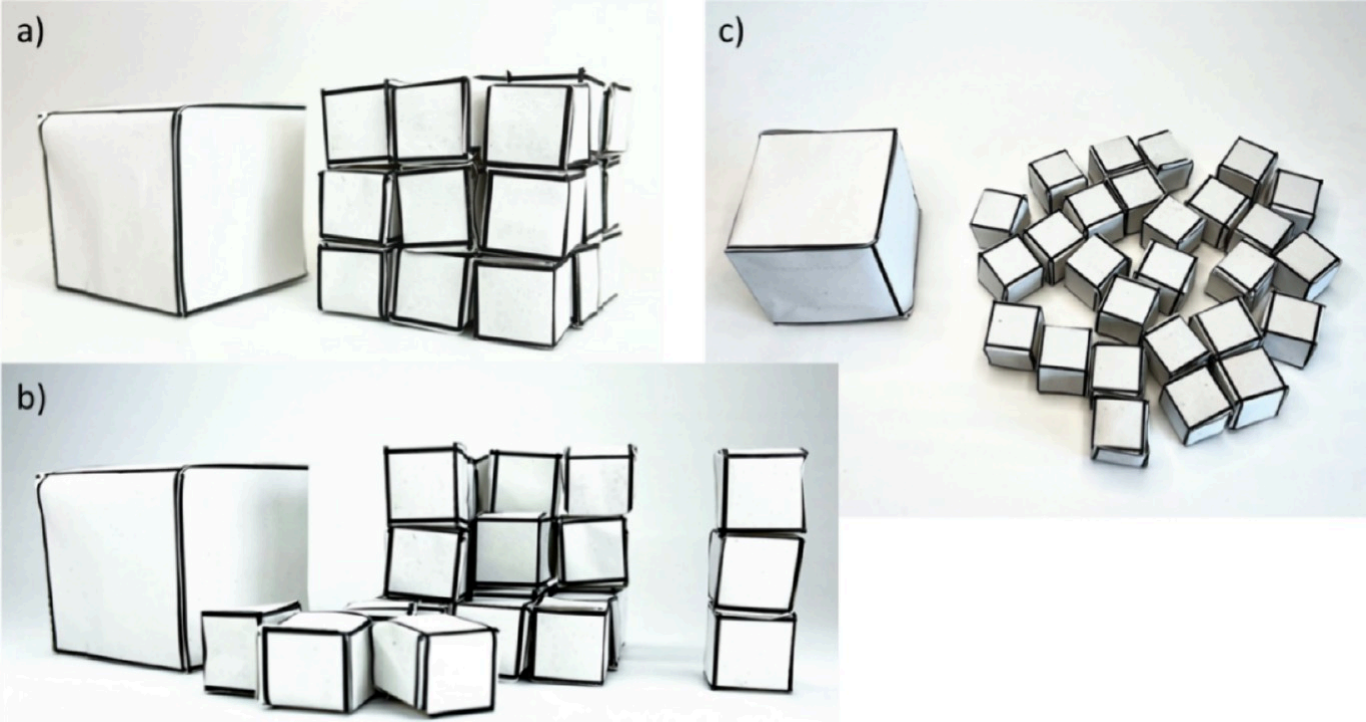
(a) 27 “small” paper cubes are assembled to form a big cube (right) and are put next to a “big” paper cube (left). (b) The 27 “small” paper cubes are disassembled (right) and put side by side with the “big” paper cube (left). (c) The surfaces of the 27 “small” paper cubes (right) and the “big” paper cube (left) can be directly compared.

The cubes are folded and glued together by paper sheets (see Figure 2, Figure S1, Video V4). It is recommended that the students do this during class: cut the cubes from the paper, fold them, and glue them. The “small” cubes are at first stacked to form a “big” cube, and then, the “big” cube is put next to it (Video V5). This shows that both cubes had the same volume. Now the assembled “big” cube is rattled and disassembled into its 27 “small” cube pieces (Video V6). The students should understand the concept of a nanomaterial. A “big” (bulk) material is graded down into small pieces of “small” (nano) material.^5^ We can take, for example, a metal, put it into a ball mill, and grind it down more and more, or we can “smash” a piece of carbon with a hammer into many smaller pieces. The material remains the same, but instead of one block of bulk material, we now have many small pieces of nanomaterial. In the example of the paper cubes, the students can see that 27 = 3 × 3 × 3 blocks of 1 cm side length have the same volume as one block of 3 × 1 cm = 3 cm side length. In order to understand the order of magnitude of this effect, the students should now try to calculate how many nanocubes of 1 nm side length one gets if one block of 1 m side length is disassembled into these nanocubes. Students should be provided with the hint that the volume of the original cube is the total volume of all of the nanocubes. One cube of 1 m side length has the volume (1 m)^3^ = 1 m^3^. One nanocube of 1 nm side length has the volume (1 nm)^3^ = (10^–9^ m)^3^ = 10^–27^ m^3^. Thus, the volume of one big cube equals the volume of 1 m^3^/10^–27^ m^3^ = 10^27^ = 1,000,000,000,000,000,000,000,000,000 nanocubes. The teacher should try to give the students a feeling of how strong this effect is.

### Variant (iii): The Use of Colors to Distinguish between “Inner” and “Outer” Surfaces

**Figure 3:**
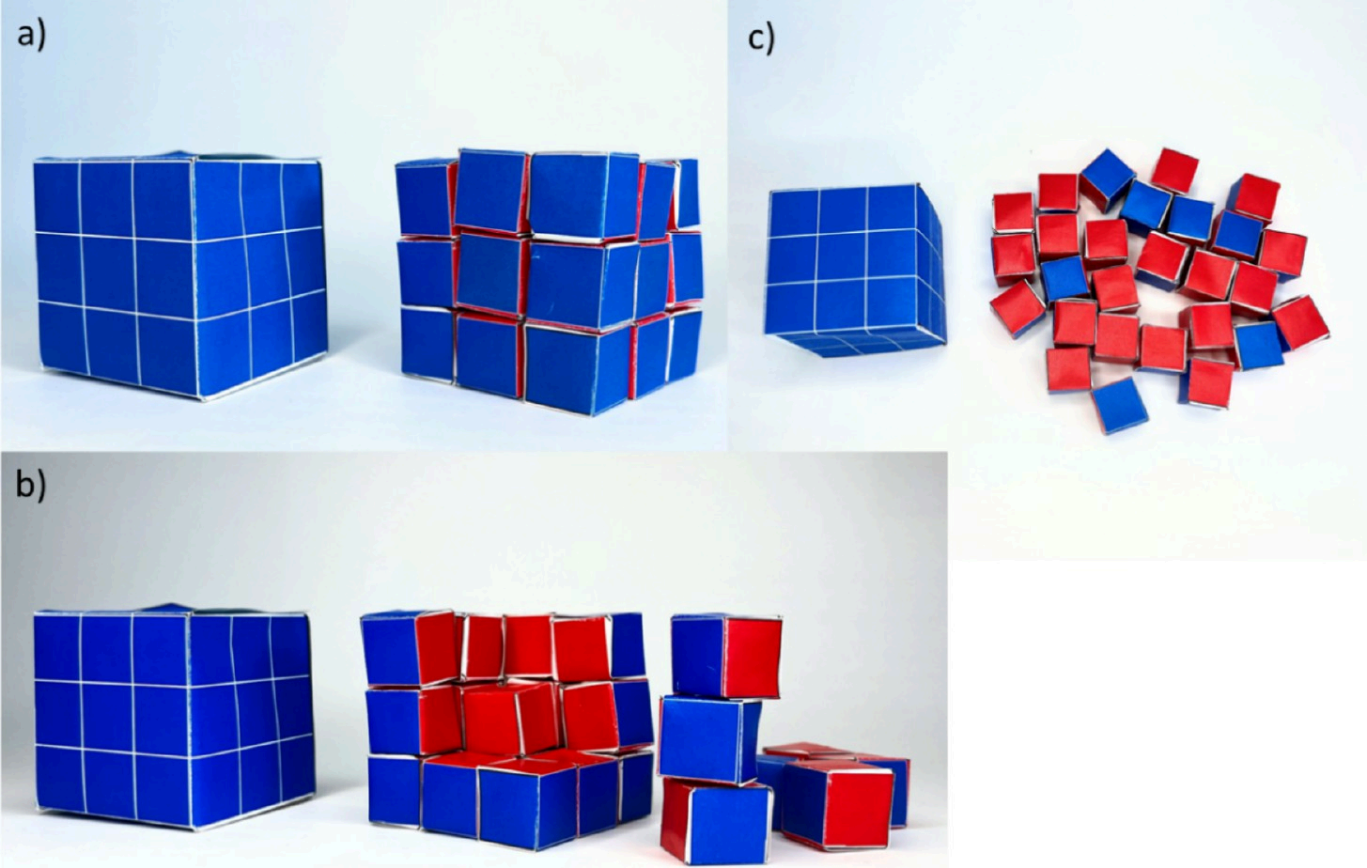
(a) The 27 colored “small” paper cubes are assembled to form a big cube with the blue faces on the outside (right) and are put next to the “big” colored paper cube (left). (b) The 27 colored “small” paper cubes are disassembled (right) and put side by side with the “big” colored paper cube (left). The appearance of the red color indicated the gained surface area. (c) The 27 colored “small” paper cubes (right) and the “big” colored paper cube (left) are compared by their colors. All red color indicates the additional surface gained by disassembly.

The cubes are folded by paper sheets, but now, the surfaces of the cubes are colored (see Figure 3 and Figure S2). The “big” cube is entirely blue, as all its sides are at the surface. The “small” cubes are blue and red in color. If assembled into a “big” cube, all the parts of the small cubes are blue are on the surface and the inner sides of the “small” cubes are red. Side by side, the “big” cube and the assembled “big” cube are all blue. However, when the assembled “big” cube is disassembled into smaller cubes, one can see by the appearance of red-colored sides how much more surface is introduced (Video V7). After having gotten a qualitative feeling about how much new surface is introduced (the red sides) by breaking down a “big” cube into 27 “small” cubes, in the next experiment, the newly introduced surface is also quantified.

### Variant (iv): Using Actual Numbers for Surface Areas for Calculating the Total Surface Area

**Figure 4:**
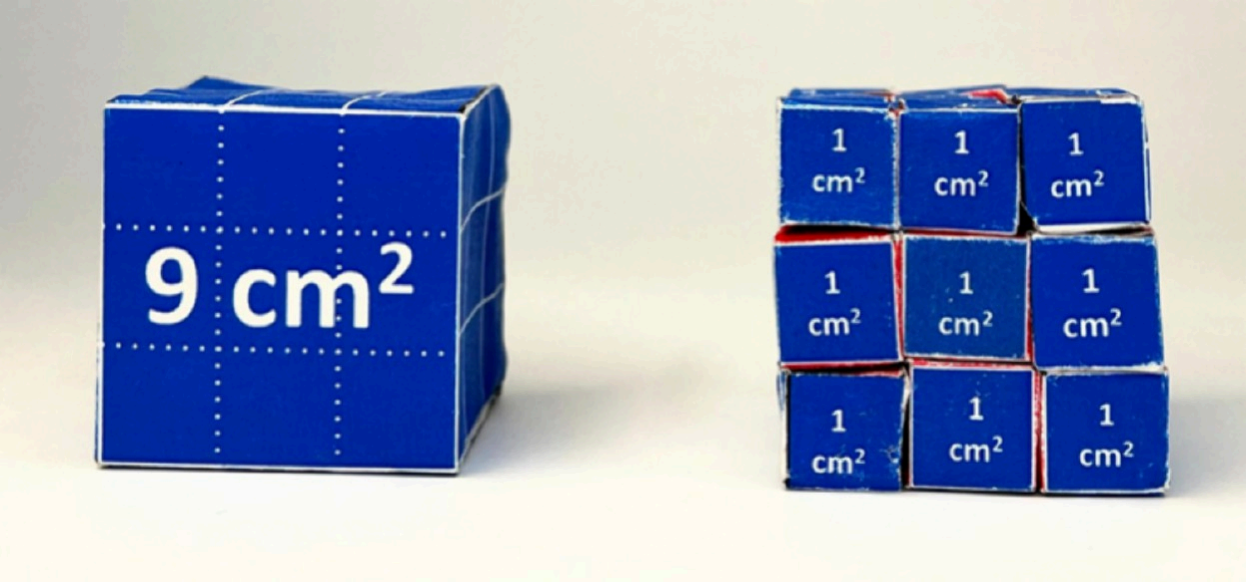
Respective surface area is printed on all of the surfaces of the “big” cube (left) and on the surfaces of the 27 “small” cubes (right).

The cubes are folded by paper sheets, but now the surfaces of the cubes had their surface area printed on them; e.g., 3 cm × 3 cm = 9 cm^2^ for the “big” cube, and 1 cm × 1 cm = 2 cm^2^ for the small cubes (see Figure 4 and Figure S3). The “big” cube and the “small” cubes, which are assembled to form a “big” cube, are put side by side, and their total surface area is read by summing up the areas printed on their surfaces (“big” cube: 6 × 9 cm^2^ = 54 cm^2^, assembled “small” cubes: 6 × 3 × 3 × 1 cm^2^ = 54 cm^2^ (Video V8)). Now the assembled “big” cube is disassembled, and the total surface from the 27 “small” cubes is calculated by summing up the areas printed on their surfaces: 27 × 6 × 1 cm^2^ = 162 cm^2^ (Video V9). Thus, by breaking down the “big” cube into 27 “small” cubes, the total surface area has increased by a factor of 162 cm^2^/54 cm^2^ = 3. The effect strongly increases when the fragments become smaller. The teacher should now ask the students to calculate how much the total surface area increases when 1 m^3^ material is broken down into 10^27^ pieces of 1 nm^3^ fragments: Surface of bulk material = 6 × (1 m)^2^ = 6 m^2^; Surface of the 10^27^ pieces nanomaterials = 10^27^ × 6 × (10^–9^ m)^2^ = 6 × 10^9^ m^2^. Thus, the total surface area increased by a factor of 6 × 10^9^ m^2^/6 m^2^ = 10^9^ = 1,000,000,000.

To summarize this part, the students should have learned that, by breaking down a material into many small (nano) pieces of material, the composition of the material remains unchanged and the mass and volume of the material remain unchanged, but the surface area of the material increases dramatically.

## Influence of the Surface-to-Volume Ratio on the Dissolution and Melting Properties of Materials

The challenge hereby is to use materials that are easily available in “small” and “big” sizes. Not everything that seems obvious at first will also work in a laboratory class. This, on the other hand, offers also the possibility to explain to the students that often secondary effects may overshadow the primary effect. We here first present variations of the experiments that are robust under laboratory class conditions and later also discuss the variants that failed and the respective reasons for this. The general concept, which students should understand, is that there are several types of chemical/physical reactions, which happen on the surface of materials, which is why the surface-to-volume ratio is of utmost importance.^6,7^

### Surface-Dependent Dissolution: The Example of Rock Sugar

**Figure 5:**
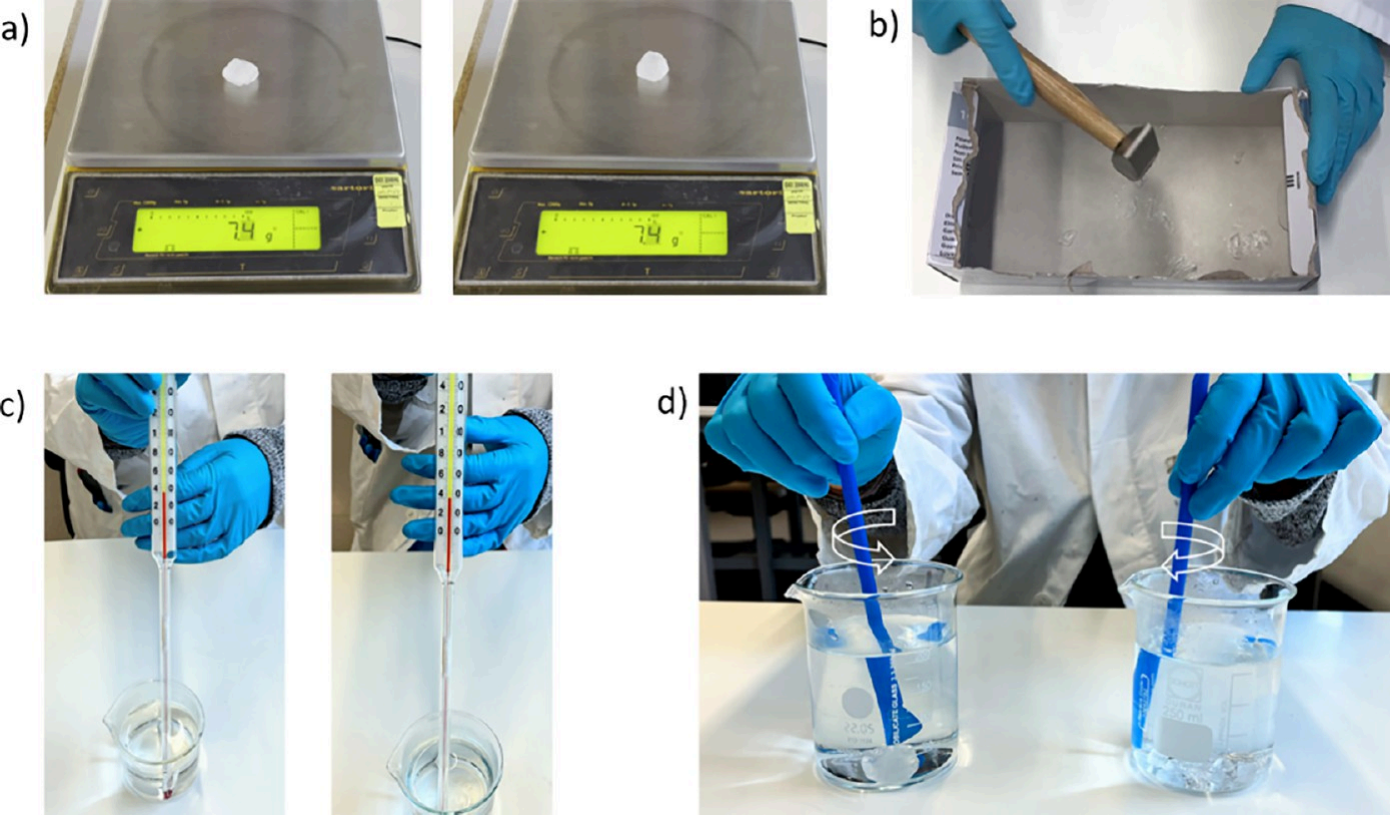
(a) The masses of two pieces of rock sugar are weighed on a balance. (b) One of the pieces is crushed using a hammer. (c) The temperature of the water in both beakers should be around 40 °C. (d) The rock sugar pieces in the water are gently stirred or shaken in order to speed up the dissolution process. The students need to observe whether the intact or crushed piece of rock sugar has dissolved first.

The first example is the dissolution of rock sugar (also commonly referred to as rock candy or sugar candy) in water. Rock sugar is composed of millimeter-sized sugar crystals and can easily be obtained from the supermarket. The process of the dissolution of rock sugar in water will be known from daily life to all students, e.g., when putting a piece of rock sugar in a cup of tea. First, the teacher should elaborate on the principle of dissolution. In solid-state (bulk) form, rock sugar is composed of crystals made of sugar molecules (note that there are many different types of sugar molecules, and the composition depends on the respective product). In the bulk of the crystal, each sugar molecule is surrounded by his or her sugar molecules. Only at the surface of the crystal do the sugar molecules also “see” the environment. Once put in water, the sugar molecules of the crystal lying at the surface are in contact with the water molecules. For a sugar molecule, it is energetically favorable to be surrounded by water molecules, instead of being surrounded by other sugar molecules. The lattice energy describes the binding energy of molecules in a crystal, i.e., how strongly the molecules stick together.^8^ The hydration energy describes the energy upon the surrounding molecules with water.^9^ For sugar, the hydration energy is greater than the lattice energy; i.e., hydration of the sugar molecules wins more energy than the breaking of molecules from the crystals costs. This is the reason sugar crystals dissolve in water. As water is only present at the surface of the crystal, dissolution happens from the surface and thus depends on the surface-to-volume ratio. As for younger students, the concept of lattice and hydration energy may be hard to grasp, the teacher can show a video about the “breaking” of sugar molecules from the surface upon the dissolution process (Video V10). Having understood the concept of dissolution, the teacher may ask the students about their guesses on what would dissolve faster in water, a “big” piece of sugar or many “small” pieces of the same total mass. After comprehending that dissolution happens from the surface, it is expected that most students will correctly answer that the “small” pieces will dissolve faster due to their higher surface-to-volume ratio. For the experimental visualization, two pieces of rock sugar will be selected that have approximately the same mass (as determined by weighing; again, relevance or experimental errors may be discussed; e.g., why it does not matter when the masses of the two pieces are not identical with the last digit; Video V11). One piece is smashed into small fragments by hitting with a hammer (Video V11). The teacher can reemphasize that the “big” piece of rock sugar and the fragmented rock sugar are the same material (“sugar”; the sugar molecules have not changed). The difference is simply the higher surface-to-volume ratio of the smashed sugar. The teacher may here refer to ball milling, which is a technical process used to fragment materials into smaller pieces (which is more “elegant” than smashing them with a hammer) and that is also used for the production of nanomaterials.^10^ Again, upon ball milling, the material remains the same, but the surface-to-volume ratio is increased. The “big” piece of rock sugar, and the fragmented sugar pieces are now put into two identical beakers with the same volume of hot water. Note that the higher the temperature of the water, the faster the dissolution process will be, but there is the risk that the students may burn themselves. We found temperatures of around 40 °C to be an appropriate choice. This temperature can be in general also obtained from the “hot water” outlet of the lab sink; i.e., no extra heating device is needed. Students can gently shake the beakers to speed up the dissolution process and observe which sugar dissolves faster (Figure 5, Video V12). The teacher may recapitulate that the high surface-to-volume ratio allows the “small” pieces of sugar to dissolve faster than the “big” piece, which is one example of why nanomaterials behave differently from their bulk counterparts.

The teacher may also want to introduce a variant for this experiment that fails and discuss the reasons for it. Here, the dissolution of a sugar loaf and the equivalent mass of sugar cubes is compared. Both can be obtained from a supermarket. The teacher then asks what students think will dissolve faster. Most likely, many students will say that this will be the sugar cubes due to the higher surface-to-volume ratio. The teacher then demonstrates the experiment. After weighing, both are put into hot water (ca. 40 °C) and their dissolution will be compared (Video V13). Surprisingly either there will be no difference or even the sugar loaf will dissolve faster, which after having discussed dissolution in terms of surface-to-volume ratio seems counterintuitive. The teacher may now involve students in a discussion about the reasons for this discrepancy. One reason might be that the density in both forms of sugar is different; e.g., the sugar cubes might be more compressed. In order to test this hypothesis, students can try to calculate the volume of the sugar loaf and the sugar cubes (the sugar loaf is assumed to be “in-between” a cone and a cylinder, and the sugar cubes are assumed as square prisms; see Figure S4). The density is the mass of the sugar (which had been determined previously by measuring its weight) divided by the respective calculated volume. In our calculation, the volume of the sugar loaf cannot be determined well, and thus the densities of the cubes and the loaf cannot be compared (Figure S4).^11^ In reality, the densities are relatively similar. The teacher may receive a variety of different arguments, which should be evaluated by model calculations as above and their relevance to explain the differences in solubility. If not mentioned, then the teacher should give the following arguments. The sugar loaf and sugar cubes are not individual sugar crystals but are composed of smaller sugar crystals (typically below 1 mm) that are pressed together. The sugar loaf and the sugar cubes are highly porous; i.e., water can immediately reach the surface of all small crystals. This means that the total surface is given by neither the surface of the cone nor the total surface of the cubes but by the surface of all small sugar crystals that are pressed together. For this reason, the argument of a higher surface-to-volume ratio of the sugar cubes versus the sugar loaf is not correct, as the surface does not describe the effective surface, which here would be the surface of all pressed-together microcrystals. With this additional experiment, the teacher can demonstrate to the students that, while often their intuitive analysis may refer to a correct effect, there are secondary effects that can change the result.

### Surface-Dependent Melting: The Example of Colored Ice

**Figure 6:**
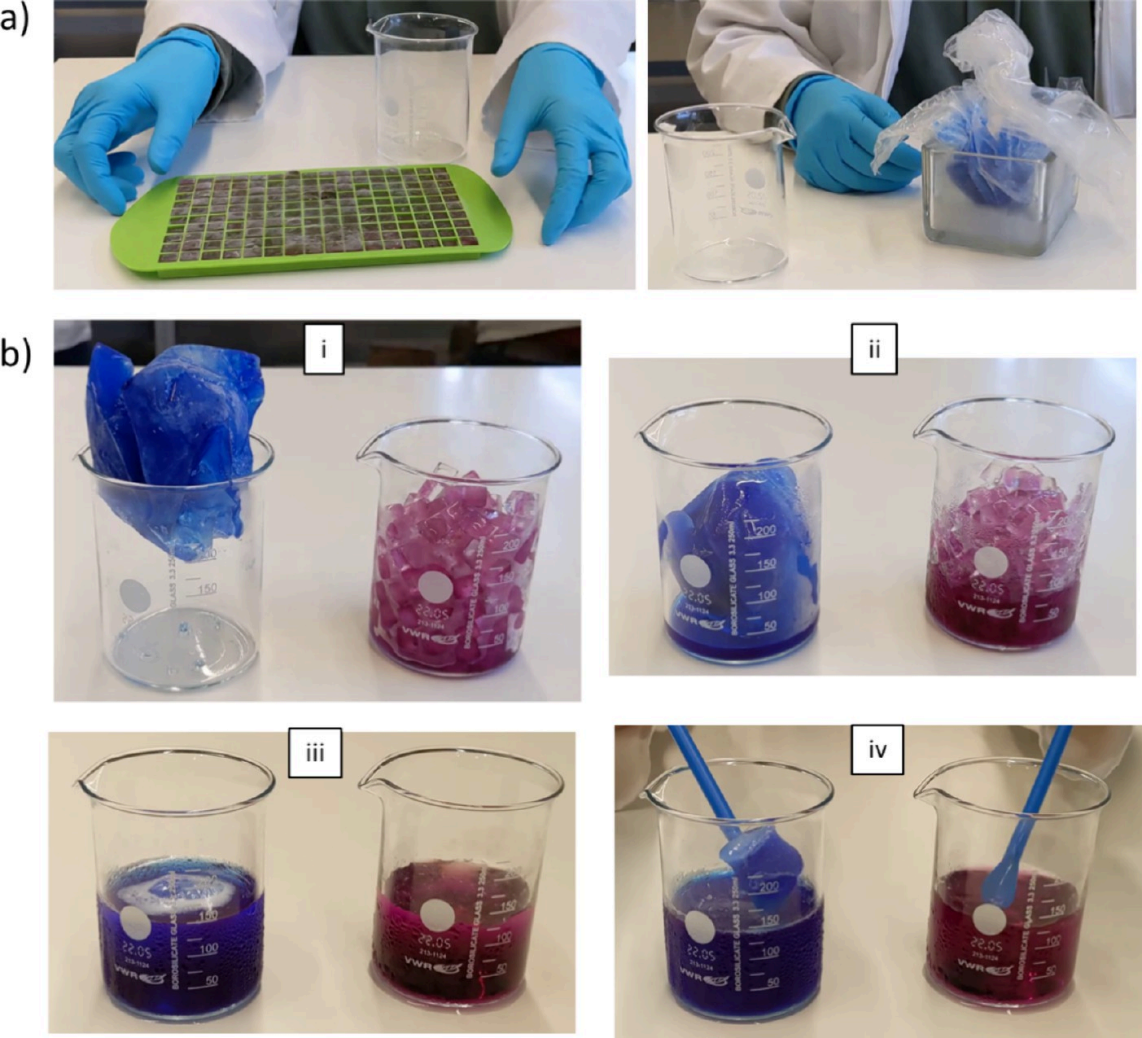
(a) An ice cube template is filled with 150 mL of red water (left) and a plastic bag is filled with 150 mL of blue water (right), which then is left overnight in a freezer. (b) The melting process of the blue big ice chunk and the red ice cubes over time at room temperature (from (i) to (iv)).

Similar to the dissolution of sugar crystals in water, also the melting of ice at room temperature is a surface-dependent effect.^12^ For this experiment, students must prepare one large block of ice and several smaller ice cubes with the same total mass. Ice cubes can be made using the ice cube templates used for cocktails. A larger block of ice can be frozen in a plastic bag. First, beakers with the same volume of water are prepared. For better visualization of the melting process, blue and red food colors can be mixed with the water before freezing. The water is then transferred in equal amounts into the ice cube template and the (transparent) plastic bag followed by freezing them in a standard household freezer (Video V14). For the experiment, the big ice block is freed from the plastic bag (simply rip the plastic bag), and the ice cubes are taken out of the cube template. Both are transferred into 2 beakers, and the speed of melting is observed (Figure 6, Video V15). The small ice cubes melt faster than the big ice block, which again can be explained by their higher surface-to-volume ratio.

To summarize this part, the students should have understood that, by fragmenting a material into smaller pieces, the molecular content of the materials remains unchanged; i.e., sugar molecules remain sugar molecules, and ice remains ice. However, the surface-to-volume ratio increases, which enhances the surface-based interaction of the material with the environment, such as dissolution and melting. Considering the drastic increase in the surface-to-volume ratio for nanomaterials, this enhanced surface-based interaction is very pronounced, which makes the nanomaterials different from their bulk counterparts.^13^

## Influence of the Surface-to-Volume Ratio on the Catalytic Properties of Materials

The catalytic properties of volume materials (in contrast to atomic/ionic catalysts) are the classic example of surface effects, as a reaction is catalyzed only in very close proximity (in general in direct contact) with the surface of the catalyst. Depending on the background knowledge of the students, the teacher first must explain the concept of catalysts. While chemistry students will be well aware of what a catalyst is, pupils in school may need a short and easy introduction. In Figure S5, a simple introduction is sketched. The “reaction” is hereby symbolized by a ball rolling down a downhill slope, driven by a reduction of its potential energy. In the case of an energy barrier (i.e., activation energy), the reaction is blocked. A catalyst can reduce the energy barrier, without itself taking part in the reaction, allowing for the reaction to start/continue.^14−16^ A classical solid state catalyst that even younger pupils will know is the catalyst used in cars, in which, in simplified words, a platinum (also other metals are used) surface as a catalyst regulates the exhaust from combustion, e.g., by oxidizing carbon monoxide into carbon dioxide.

### Colorimetric Assays to Observe Catalytic Reactions by the Naked Eye

**Figure 7:**
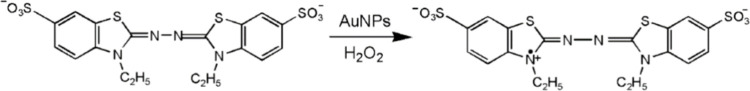
Oxidation reaction of 2,2′-azino-bis(3-ethylbenzothiazoline-6-sulfonic acid) diammonium salt (ABTS) catalyzed by gold surfaces. Image adapted with permission from Wang et al.^18^ Copyright 2012 Wiley-VCH.

There are catalytic reactions that can be conveniently followed by colorimetric assays. In such a reaction, a colored or colorless reactant is converted upon the presence of the catalyst in a colorless or colored product, respectively. Here, we suggest using the oxidation of dissolved colorless 2,2′-azino-bis(3-ethylbenzothiazoline-6-sulfonic acid) diammonium salt (ABTS)^17^ to the respective green translucent oxidized version in the presence of an oxidizing reagent (in this case H_2_O_2_) and a gold (Au) surface as catalyst^18−20^ (Figure 7):
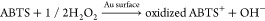


Only in the presence of enough Au surface as a catalyst can the ABTS be oxidized, and the solution turns a green color. The most straightforward comparison would be to carry out the reaction on the surface of a gold foil (low surface-to-volume ratio), in contrast to gold nanoparticles (NPs; high surface-to-volume ratio). Gold foils are available in the form of gold leaves (in German: Blattgold) used for food decoration. Attempts to use gold leaves versus gold NPs (at equal Au elemental concentration as determined with inductively coupled mass spectrometry (ICP-MS)) as catalyst for the oxidation of ABTS however failed in the sense that the color change was almost immediate after adding the gold foil. There are two reasons for the failure of the experiment. The first one is that it is almost impossible to place the same amount of gold in both reactions. Even placing the smallest amount possible of gold foil will still lead to more than the amount of gold in a colloidally stable nanoparticle solution. The second reason is that the Au surfaces, in general, are “occupied”. In the case of the Au NPs, their surface is covered by ligands^21,22^ (see Figure S6), whereas in the case of the gold leaves, it is unknown what organic material might be present on their surface during the production and packaging processes. The ligand coating slows down the catalytic reaction due to blockage of the reaction sites^18,23,24^ (see Figure S7). The teacher may discuss this problem in general, leading to the conclusion that for a “fair” comparison the gold surface needs to have the same potential “organic contamination” on its surface.

### Visualization of the Catalytic Properties in Dependence of the Total Surface Area

A good comparison can thus be made by Au NPs that have been synthesized by the same method, i.e., stabilized by the same organic ligands on their surface. While there are many excellent protocols available for synthesizing Au NPs of different sizes,^25,26^ we propose the use of commercial citric acid stabilized Au NPs (BBI Solutions, 15 nm Au NPs EM.GC15 and 200 nm Au NPs EM.GC200), so that this experiment can be carried out in schools without a chemistry laboratory. Citric acid is a weak ligand and thus will not block the catalytic activity too much. The teacher should start by questioning students how the catalytic activity of “small” and “big” Au NPs can be best compared concerning the respective amounts of Au NPs that should be used in the catalytic reaction. In a catalyst, the inner part does not contribute to the catalytic activity (only the surface does), but it contributes to weight, which is relevant in terms of costs (noble metals such as Pt are pricey), and reduced weight is for example wanted in car catalysts. For a direct comparison, the mass of the Au NPs in solution should be the same for both NP sizes. The teacher can give an exercise to the students to calculate the mass *m*_NP_ of one NP of diameter *d*_NP_ assuming the NP as a perfect sphere (using the bulk density of Au of ρ_Au_ = 19.3 g/cm^3^):
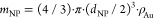
For *d*_NP_ = 15 and 200 nm Au NPs, this results in *m*_NP_ = 0.034 and 80 fg, respectively. The total mass *m*_Au_ of Au in solution depends on the number of *N*_NP_ of NPs in solution:
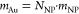
For *d*_NP_ = 15 nm Au NPs, there must be more NPs added to solution *N*_NP(15 nm)_ than for 200 nm Au NPs in order to have the same mass of Au in solution:
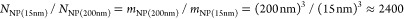
The Au NP *c*_NP_ concentration of the 15 nm Au NPs thus should be around 2400 times higher than the concentration of the 200 nm Au NPs.

Note that, for the oxidation of ABTS, H_2_O_2_ is needed. For security reasons, the teacher should carry out the experiment or, in the case students are conducting the reaction, take special care of security, which in particular involves proper personal protection such as the use of goggles and gloves.

**Figure 8:**
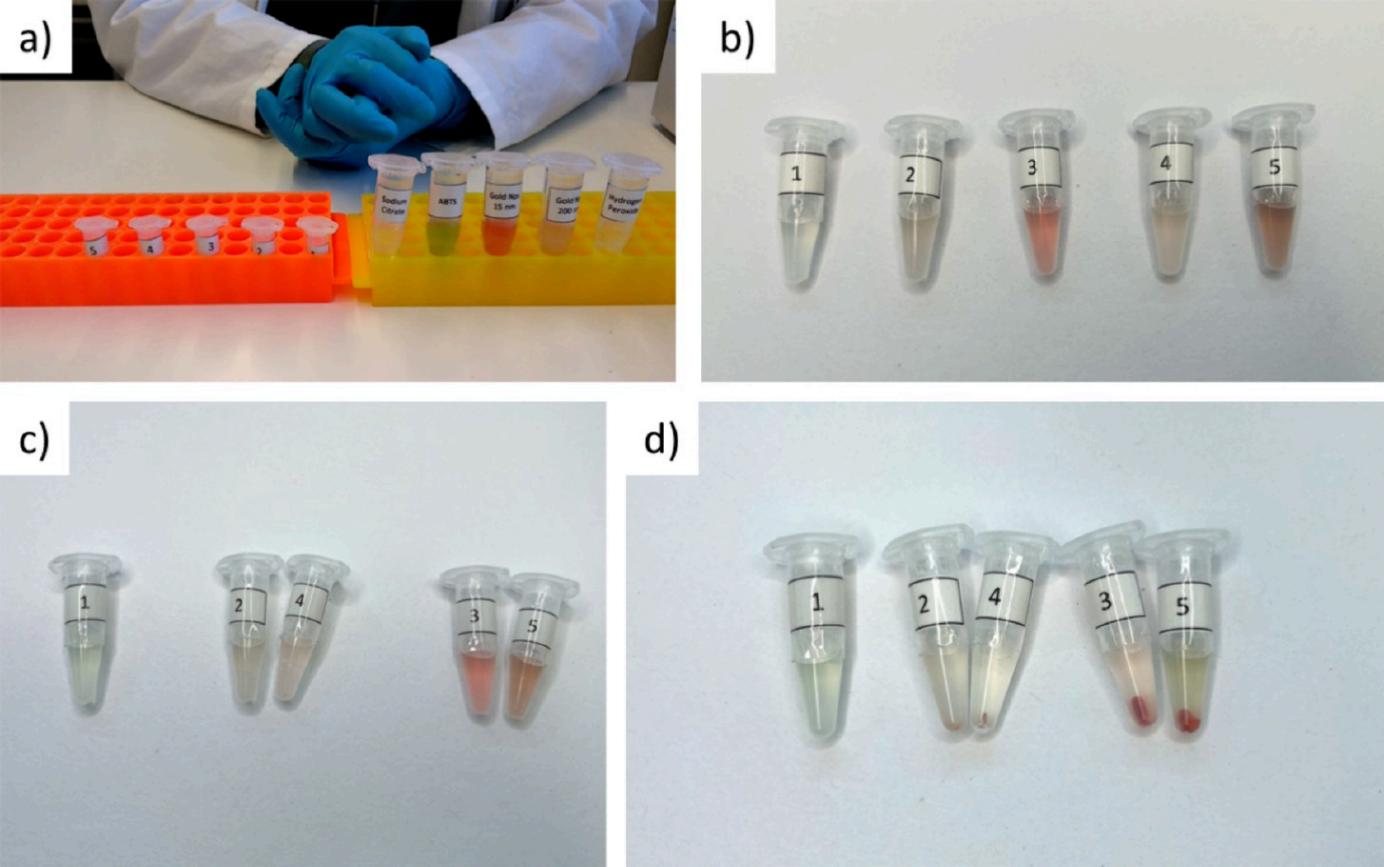
(a) Five Eppendorf tubes are prepared for the reaction (orange rack) in addition to the following stock solutions: sodium citrate (0.01 M, pH= 4), ABTS (20 mM), solutions of 15 nm (2.86 nM) and 200 nm Au NPs (1 pM), and H_2_O_2_ (2 M) (yellow rack). Photos of the solutions of reactions #1–#5 (b) immediately after preparation of the reaction solutions and (c) after 20 min at 37 °C. (d) Reaction after centrifugation at 21,000*g* for 15 min.

In Figure 8, the reaction is outlined. The teacher prepares 5 Eppendorf tubes in which the reactions are carried out and prepares stock solutions of sodium citrate (0.01 M, pH= 4) as buffer, H_2_O_2_ (9.8 M), ABTS (20 mM), and solutions of 15 nm (2.86 nM) and 200 nm (1 pM) of Au NPs (Figure 8a). Note that the commercially available 15 nm Au NPs solution is bought with a higher concentration than the one used for this experiment. Its concentration is 2.5 μM. Therefore, it must be diluted to reach the desired concentration of 2.86 nM. On the other hand, the 200 nm Au NPs solution is already bought with the correct concentration.

The teacher should explain to the students that for such a reaction appropriate controls are always needed. The oxidation of ABTS can happen only when both Au NPs and H_2_O_2_ are present. Solutions without Au NPs or H_2_O_2_ are, therefore, essential controls. Here, 5 different reactions are prepared (Table 1, Figure 8b, Video V16):
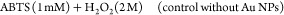
1
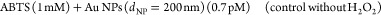
2
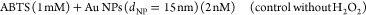
3
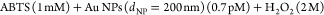
4
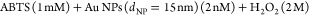
5

**Table 1:** Summary of Volume to Take from the Stock Solutions Sodium Citrate Buffer (0.01 M, pH = 4), H_2_O_2_ (9.8 M), and ABTS (20 mM) and Solutions of 15 nm (2.86 nM) and 200 nm (1 pM) Au NPs to Reach the Desired Concentrations of H_2_O_2_ (2 M), ABTS (1 mM), and Solutions of 15 nm (2 nM) and 200 nm (0.7 pM) Au NPs in a Final Volume of 500 μL

reaction	ABTS	Au NPs	H_2_O_2_	sodium citrate buffer	final volume
1	25 μL		102 μL	373 μL	500 μL
2	25 μL	350 μL		125 μL	500 μL
3	25 μL	350 μL		125 μL	500 μL
4	25 μL	350 μL	102 μL	23 μL	500 μL
5	25 μL	350 μL	102 μL	23 μL	500 μL

The reaction is carried out for 20 min at around 37 °C (lower and higher temperatures will decrease and increase the catalytic rate, respectively). As the Au NPs provide a reddish color to the solution, the green color of oxidized ABTS would be hard to see with the naked eye (Figure 8c). Therefore, at the end of the reaction, the samples are centrifuged to precipitate the Au NPs at the bottom of the flask, leaving the supernatant in the color green yielded by the reaction. In Figure 8d, it can be seen that there is no (sufficient) production of oxidized ABTS in the case of the controls #1, #2, and #3, as the solution did not turn green. Also, in the case of the 200 nm Au NPs in the presence of H_2_O_2_ (#4), no reaction can be observed. Only in the case of the 15 nm NPs in the presence of H_2_O_2_ (#5) did the solution turn green. This shows that, due to their higher surface-to-volume ratio, the 15 nm Au NPs not the 200 nm Au NPs (at the same elemental Au concentration) could oxidize ABTS.

To summarize this part, the students should have understood that the catalytic reaction depends on the available surface of the catalyst. In this way, due to their higher surface-to-volume ratio, small NPs are in general better catalysts than large particles (at the same total mass, provided the same or similar surface coating).

## Conclusions

There are many other ways to demonstrate surface-to-volume-ratio-dependent reaction properties of materials. Another example would involve covering small and large cubes with double-stick tape and rolling them over powder, which would stick to their surface. However, in our experience, melting and catalysis are the most suited ones. Melting is a process that is known to students of all ages and can be easily demonstrated. Catalysis involves more effort to demonstrate but has an extremely high practical relevance and should especially attract the attention of the students. The teacher should use this experiment to talk about the potential and risks of nanomaterials. Catalysts are highly important for technical applications, as already mentioned in catalytic converters in cars. If platinum in the nanostructured form is used, compared to the same mass of bulk Pt, a much higher catalytic performance is achieved, which allows for reducing weight. On the other hand, risks should also be mentioned. As demonstrated, the surface of small Au NPs has catalytic properties, which in fact may generate reactive oxygen species (ROS), which are toxic.^27^ In contrast to bulk gold, nanoparticulate gold can exhibit toxic effects. This can be seen in vitro in cell cultures, where cells exposed to Au NPs show reduced viability.^28,29^ Long-term exposure to Au NPs may also result in harmful in vivo long-term toxicity.^30,31^ Making materials smaller enhances their reactivity, which can be harnessed, but also involves some potential risks. The nanonature of materials is thus an important property. It does not make much sense to ask the question of whether Au NPs are toxic, as this will not only depend on the element (i.e., Au) but also on the size (and surface coating) as well as the contact route.

We have performed these experiments with students from ages of around 10 (no calculations) up to adults (bar meetings like the public series “Physik vom Fass”),^32^ as with chemistry and physics students. It is important to adjust the presentation according to the level of knowledge of the students. For younger pupils, the “construction” part of gluing together the cubes has turned out to receive the best attention, but quantitative calculations should be left out. For advanced students, quantitative evaluation of the melting and catalysis parts is suggested. In the Supporting Information T1, a document is provided which suggests which experiments can be best carried out with different age groups.

## Supporting Information Available

Video V1: construction and assembly of nanocubes (MP4)
Video V2: construction and assembly of nanocubes (MP4)
Video V3: construction and assembly of nanocubes (MP4)
Video V4: construction and assembly of nanocubes (MP4)
Video V5: construction and assembly of nanocubes (MP4)
Video V6: construction and assembly of nanocubes (MP4)
Video V7: construction and assembly of nanocubes (MP4)
Video V8: construction and assembly of nanocubes (MP4)
Video V9: construction and assembly of nanocubes (MP4)
Video V10: dissolution of sugar (MP4)
Video V11: melting of ice blocks of different individual volumes (MP4)
Video V12: melting of ice blocks of different individual volumes (MP4)
Video V13: melting of ice blocks of different individual volumes (MP4)
Video V14: melting of ice blocks of different individual volumes (MP4)
Video V15: melting of ice blocks of different individual volumes (MP4)
Video V16: catalytic reaction (MP4)
Figures S1–S3: Folding patterns for constructing paper cubes; Figure S4: Volume determination of sugar loaf; Figure S5: Sketch of catalytic reaction; Figure S6: Sketch of gold nanoparticle; Figure S7: Sketch about the influence of the ligand coating of nanoparticles on their catalytic surface activity; Text T1: Documents in which a selection of the experiments for different age groups is provided, together with suggestions for the assessment of the learning success (PDF)
