# Supplementary material for: Visualization of the High Surface-to-Volume Ratio of Nanomaterials and Its Consequences
Source: J Chem Educ. 2024 Jul 3;101(8):3146–55. doi: 10.1021/acs.jchemed.4c00089 (PMC11328123; doi:10.1021/acs.jchemed.4c00089)
Supplement: Supplementary file 17 — ed4c00089_si_017.pdf [file ed4c00089_si_017.pdf]

Supporting Information

Visualization of the High Surface-to-Volume Ratio of Nanomaterials and its Consequences

Maria Pozzi[1], Sarodi Jonak Dutta[1], Mia Kuntze[1,2], Jeannette Bading[1], Johanna S. Rüßbült[1], Cornelius Fabig[1], Malte Langfeldt[1], Florian Schulz[1], Patricia Horcajada[3], Wolfgang J. Parak[1,*]

[1]Fachbereich Physik, Universität Hamburg, 22607 Hamburg, Germany
[2]Ratsgymnasium Rotenburg, 27356 Rotenburg, Germany
[3]Advanced Porous Materials Unit, IMDEA Energy Institute, 28935 Móstoles-Madrid, Spain
[*]corresponding author: wolfgang.parak@uni-hamburg.de

Supporting Figure S1: Layout of paper to print for cutting-out and folding paper cubes

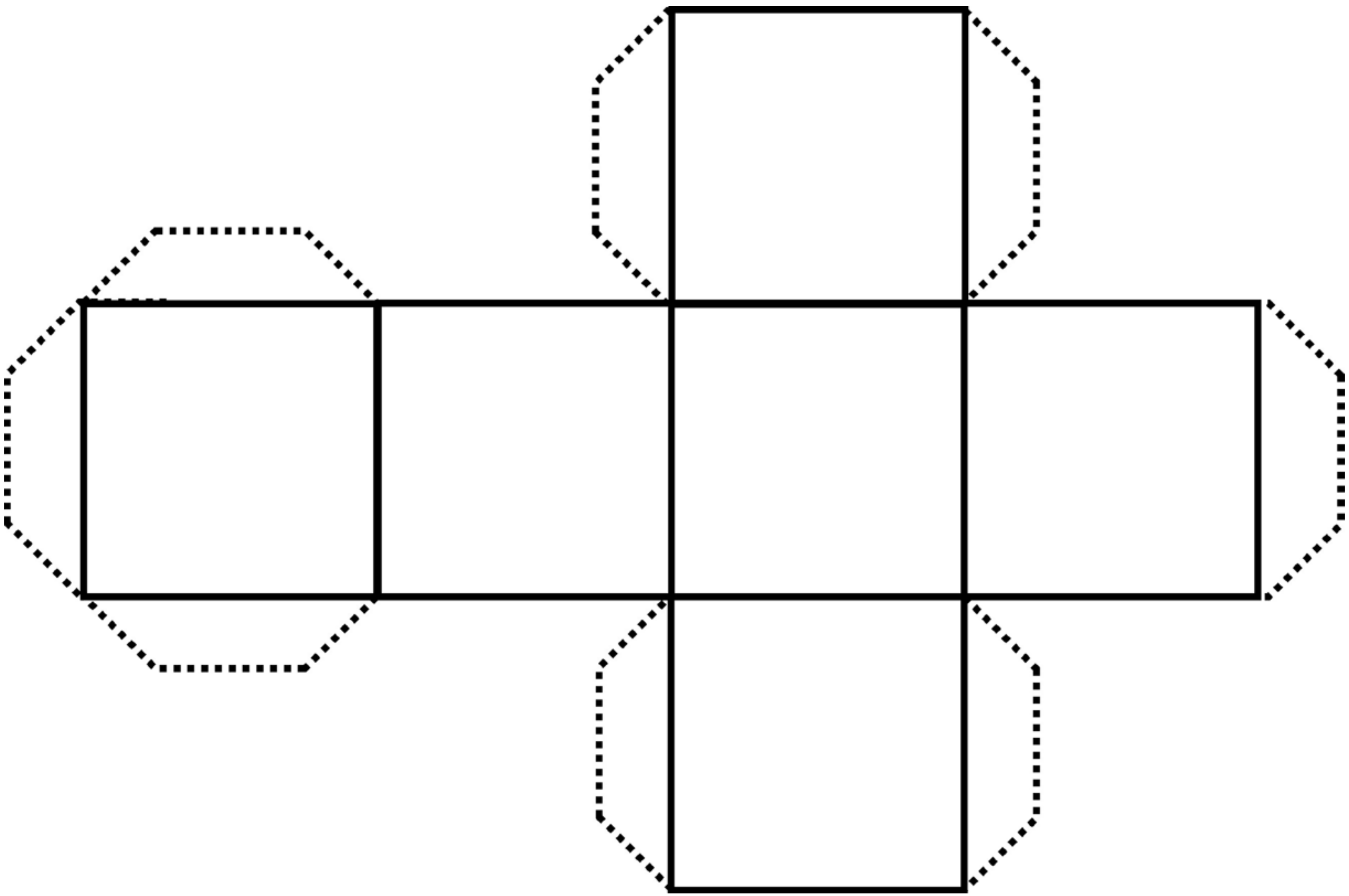

Supporting Figure S1: Layout of paper to print for cutting-out and folding paper cubes

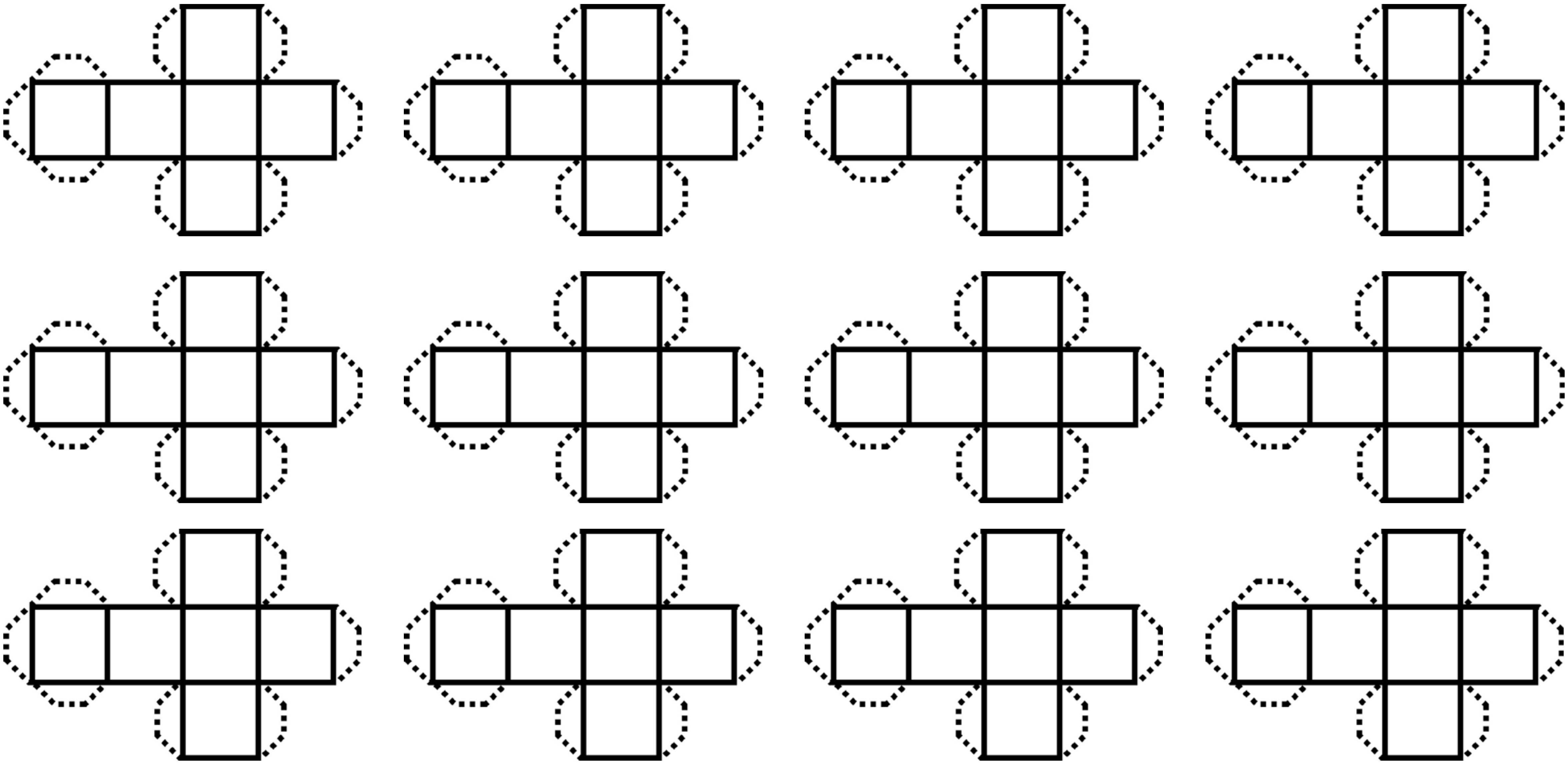

Supporting Figure S1: Layout of paper to print for cutting-out and folding paper cubes

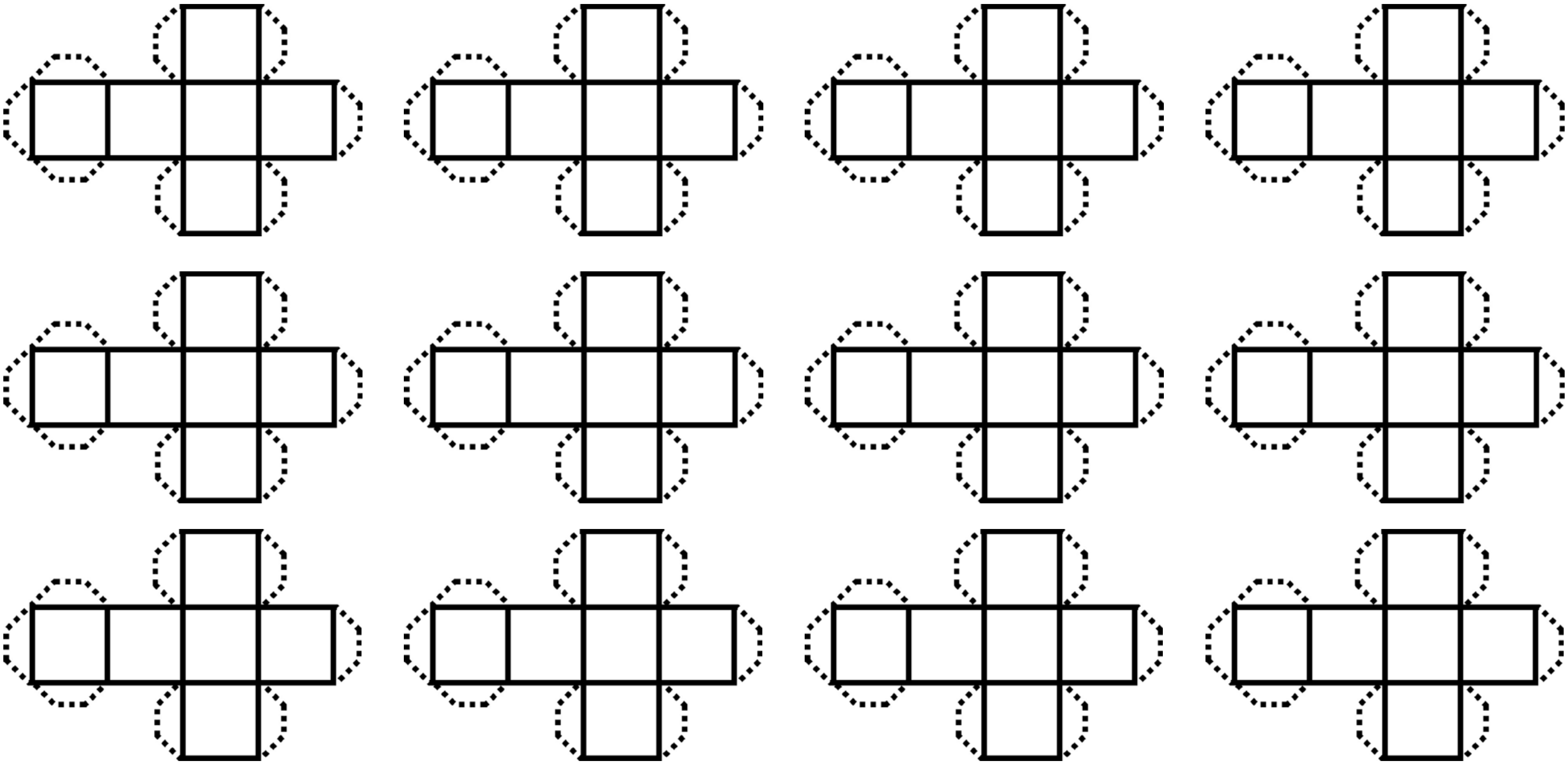

Supporting Figure S1: Layout of paper to print for cutting-out and folding paper cubes

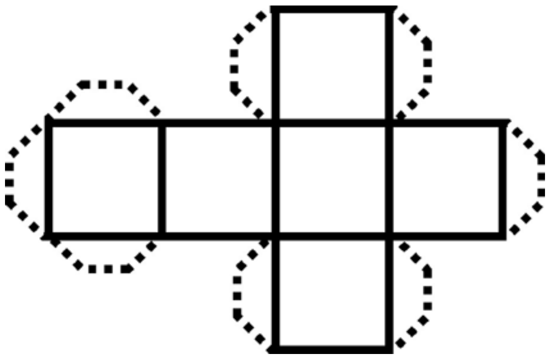

Supporting Figure S2: Layout of paper to print for cutting-out and folding colored paper cubes

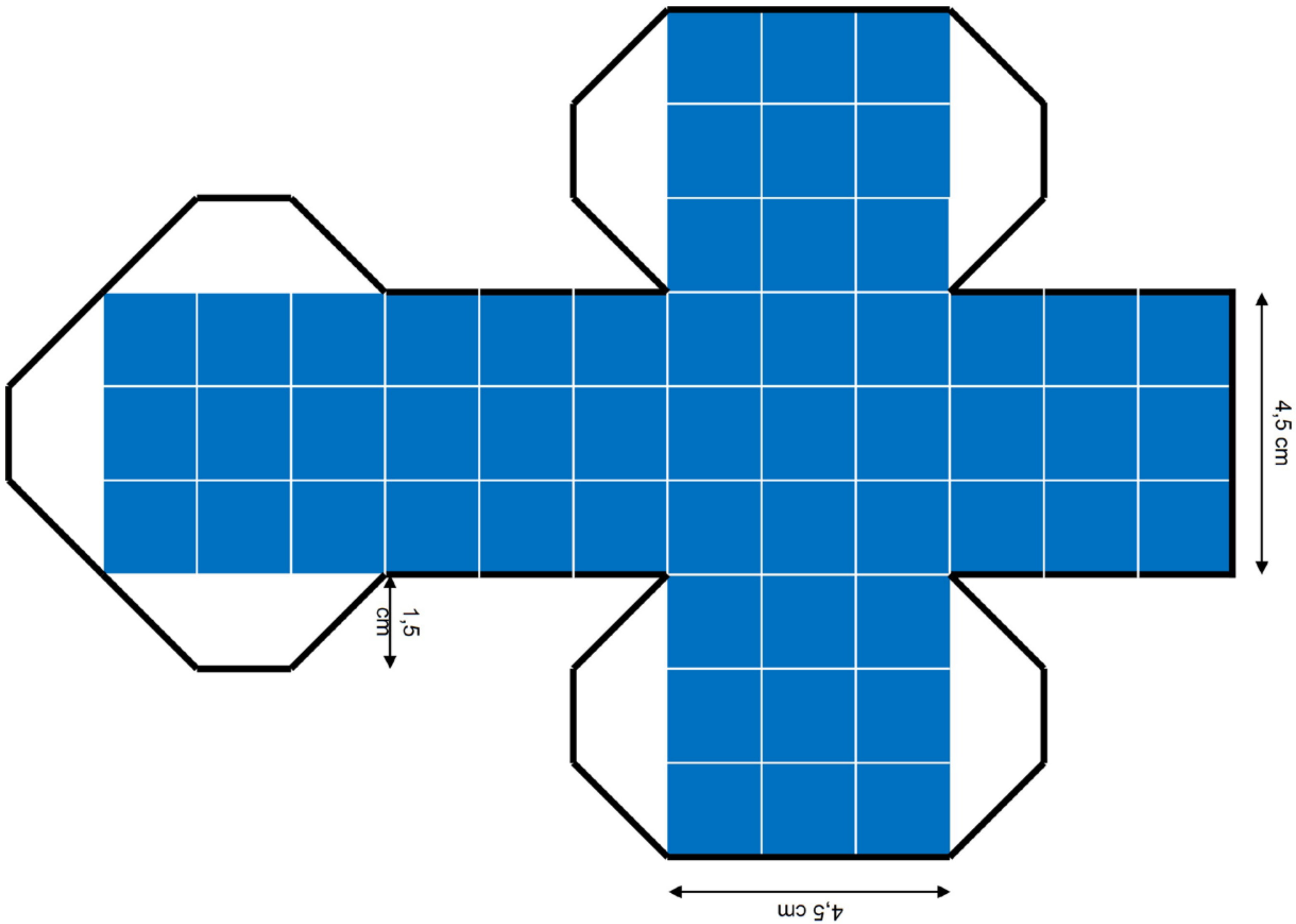

Supporting Figure S2: Layout of paper to print for cutting-out and folding colored paper cubes

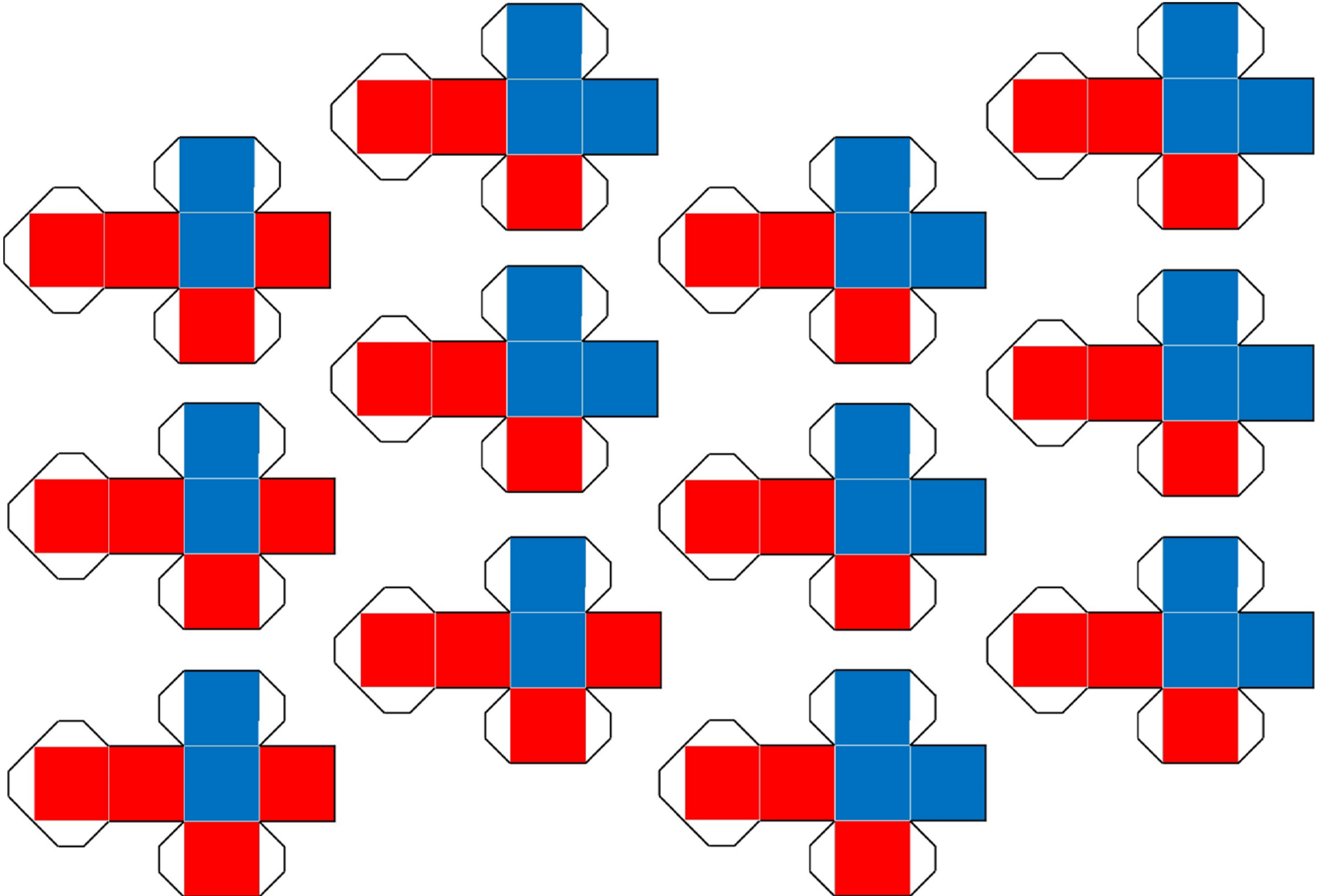

Supporting Figure S2: Layout of paper to print for cutting-out and folding colored paper cubes

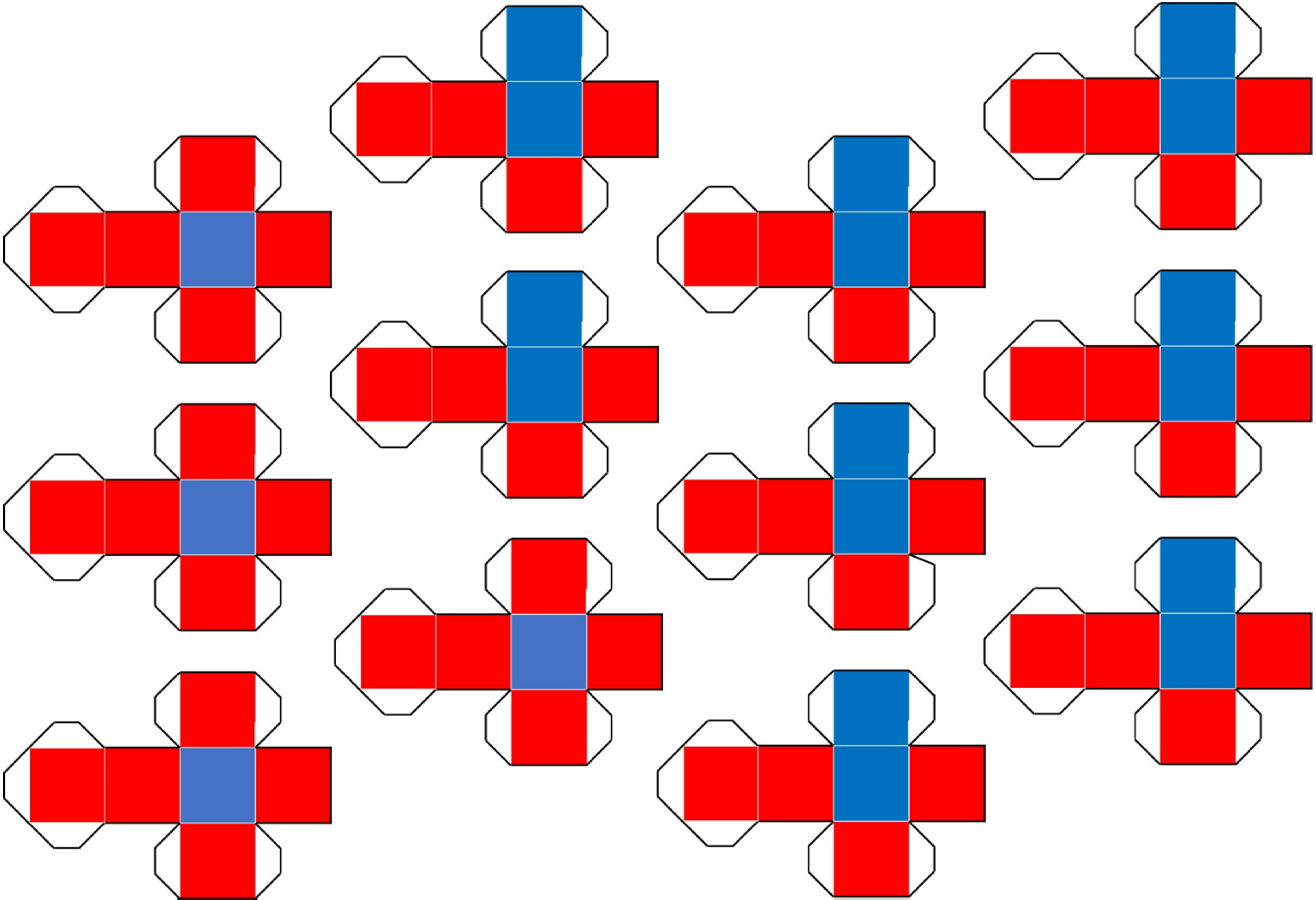

Supporting Figure S2: Layout of paper to print for cutting-out and folding colored paper cubes

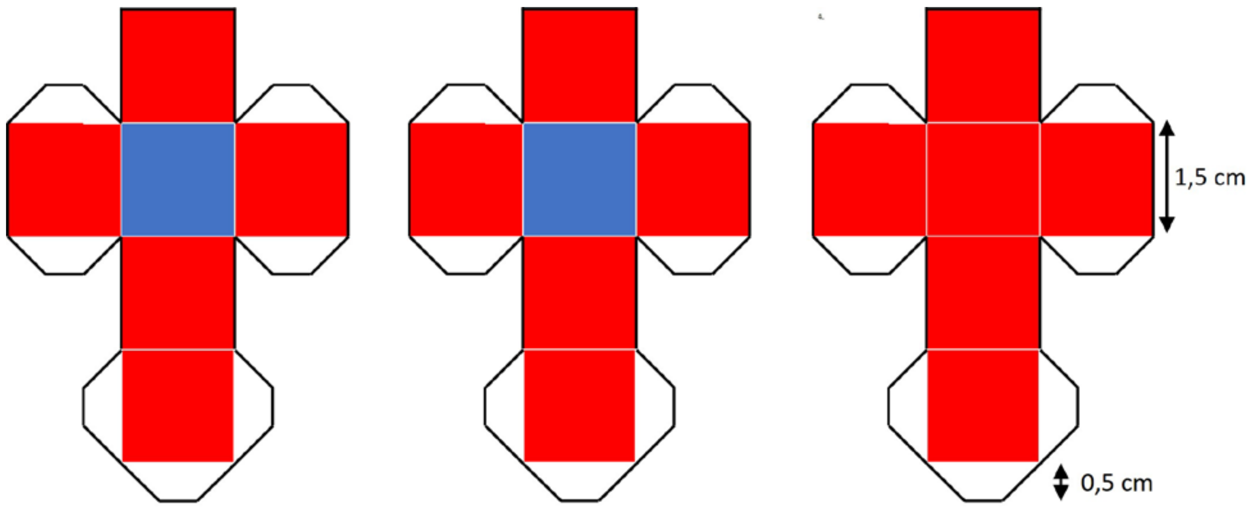

Supporting Figure S3: Layout of paper to print for cutting-out and folding colored and numbered paper cubes

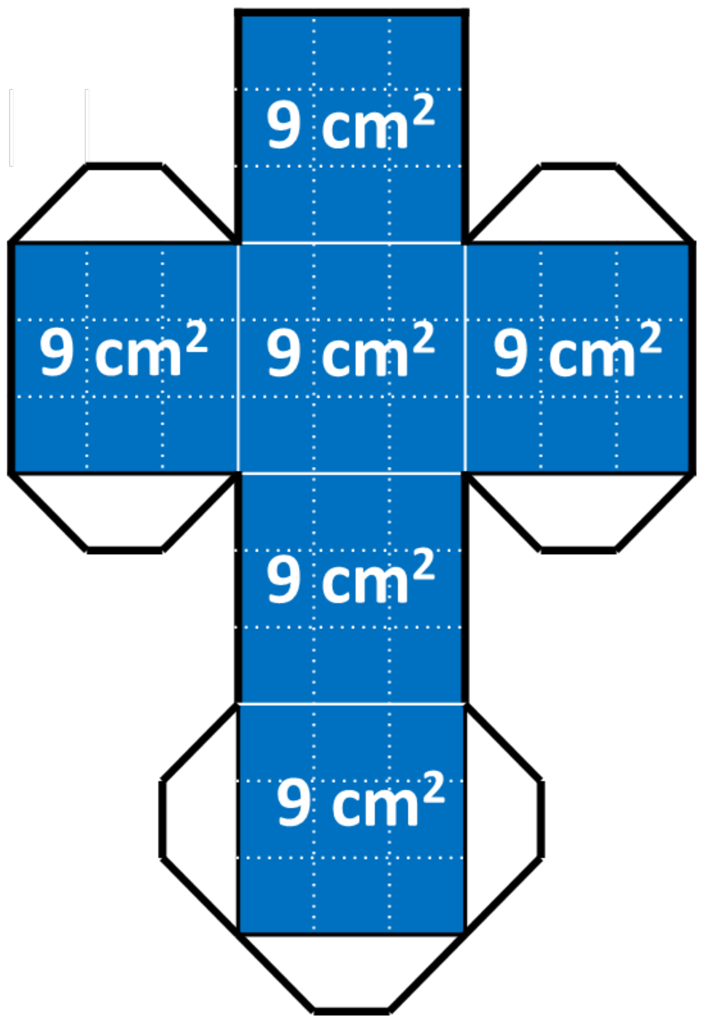

Supporting Figure S3: Layout of paper to print for cutting-out and folding colored and numbered paper cubes

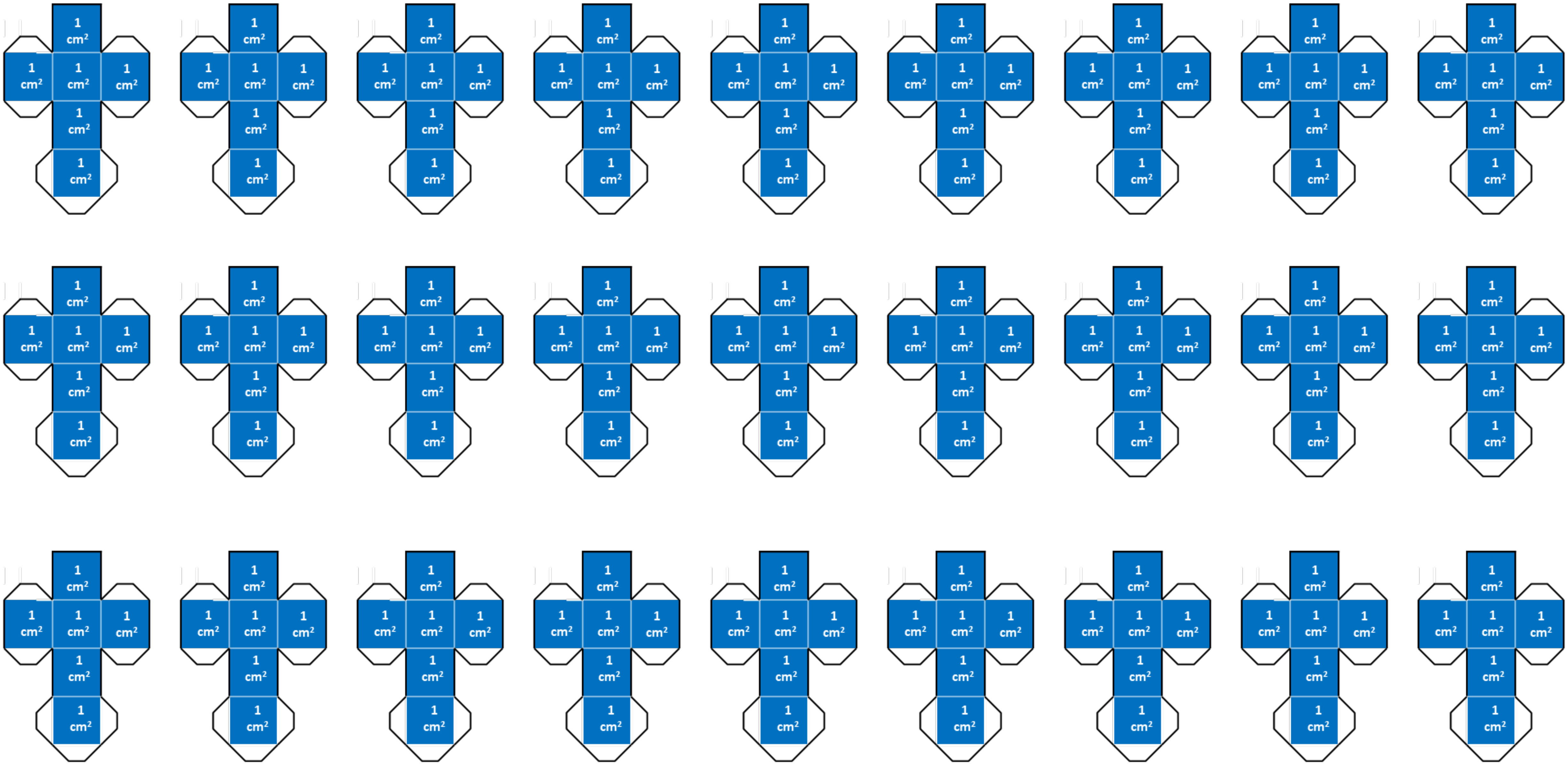

Supporting Figure S4: Calculation of the density of a sugar loaf and of a sugar cube

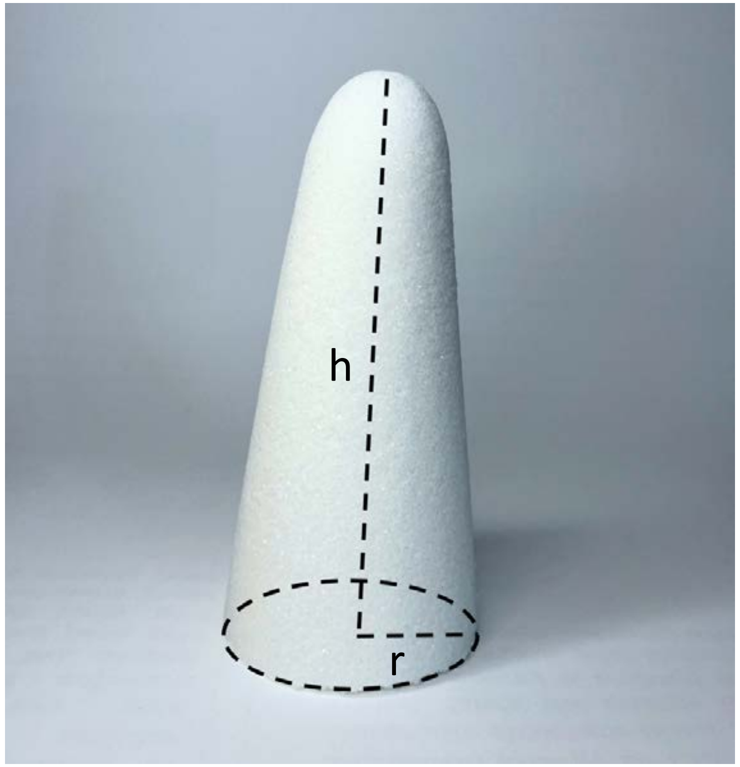

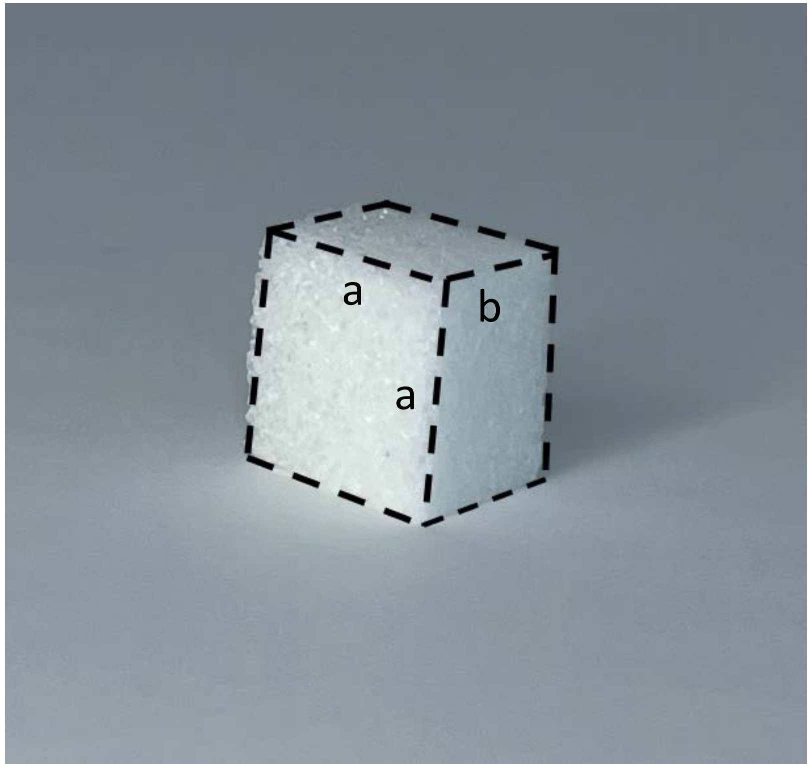

The sugar loaf is assumed somewhere between a cone and a cylinder

Cone:
$r = 3.4$ cm, $h = 13.2$ cm
Volume $V = (1/3)\cdot\pi\cdot r^2\cdot h \approx 159.8$ cm^3
Mass $m = \rho\cdot V = 250.8$ g
$\Rightarrow \rho = m/V \approx 1.57$ g/cm^3

Cylinder:
$r = 3.4$ cm, $h = 13.2$ cm
Volume $V = \pi\cdot r^2\cdot h \approx 479.4$ cm^3
Mass $m = \rho\cdot V = 250.8$ g
$\Rightarrow \rho = m/V \approx 0.52$ g/cm^3

The sugar cube is assumed as a square prism

Square prism:
$a = 1.6$ cm, $b = 1.2$ cm
Volume $V = a^2\cdot b \approx 3.072$ cm^3
Mass $m = \rho\cdot V = 3.64$ g
$\Rightarrow \rho = m/V \approx 1.18$ g/cm^3

Supporting Figure S5: Working principle of a catalyst

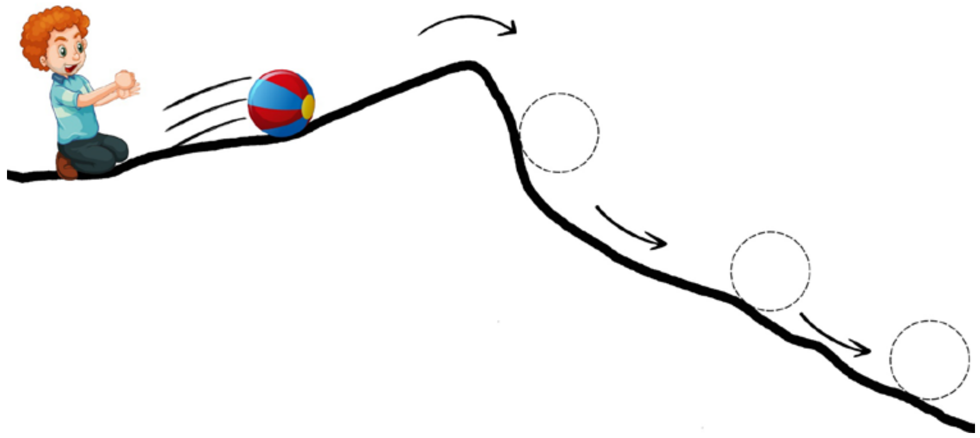

the „reaction“ (here a rolling ball rolling down a slope) is blocked by a barrier

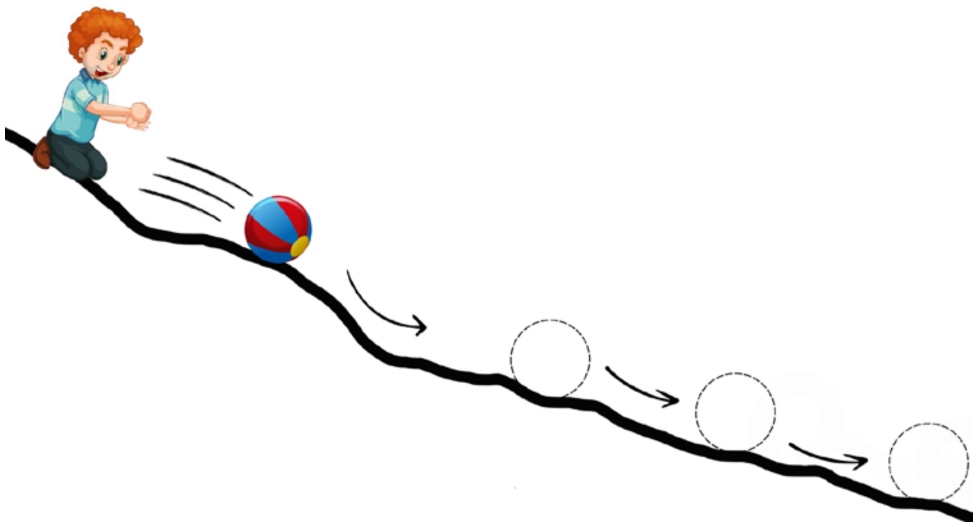

presence of the catalyst lowers the barrier and the „reaction“ can take place

Supporting Figure S6: AuNPs covered by ligands

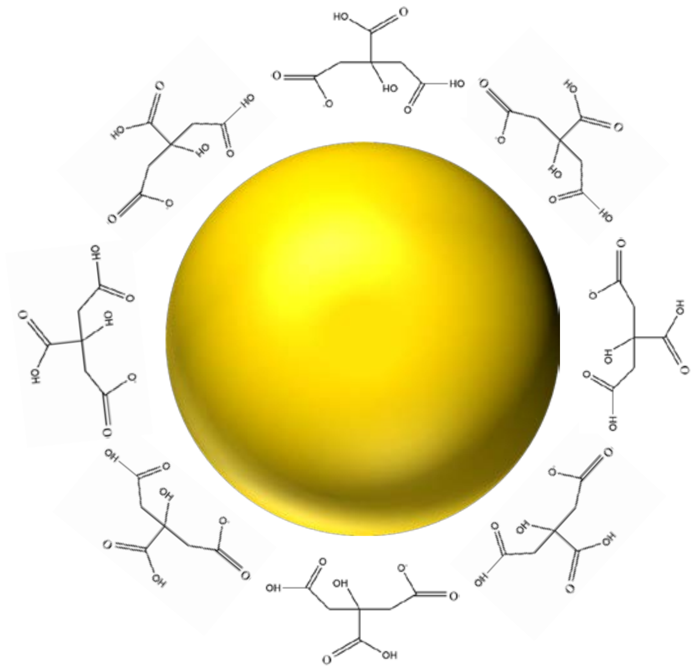

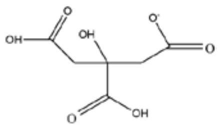

Citric Acid

Supporting Figure S7: The ligand coating slows down the reaction

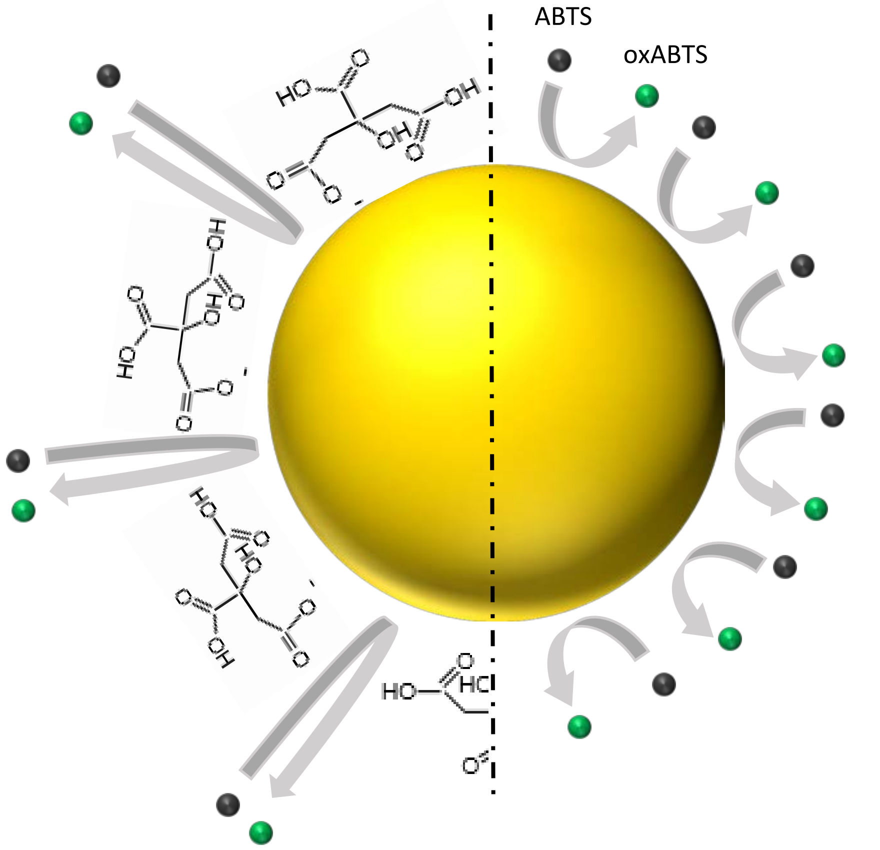

2,2’-azino-bis(3-ethylbenzothiazoline-6-sulfonic acid) diammonium salt (ABTS)

Oxidazed 2,2’-azino-bis(3-ethylbenzothiazoline-6-sulfonic acid) diammonium salt (oxABTS)

Supporting Text T1: documents in which a selection of the experiments for different age groups is provided, together with suggestions for the assessment of the learning success

experiment	young pupils (10 - 15 years)<br><br>estimated time: 4 hours	older pupils (16- years) and undergraduate students<br><br>estimated time: 6 hours	general public<br><br>estimated time: 1 hour
Introduction - how to visualize the increase in surface-to-volume ratio	• perform variant ii and variant iii; the pupils should actually glue the paper cubes together<br>• skip the calculations<br>• assessment: did the pupils understand that by cutting down materials the volume stays the same while the surface increases?	• perform variant i, variant iii, and variant iv; upon availability of time the cubes can already be pre-prepared by the teacher; limit the time for the gluing together of the cubes at any rate<br>• perform the calculations<br>• assessment: by cutting down a cube into 9 similar cubes, how much does the surface-to-volume-ratio increase?	• show variant i<br>• alternatively you can take an object from daily life, such as an avocado: cut it with a knife into small pieces and explain that while the mass of the avocado remained constant, the pieces possess a higher surface<br>• skip the calculations<br>• assessment: ask the question whether the audience understood that cutting down a material into smaller pieces increases the surface-to-volume-ratio
Influence of the surface-to-volume ratio on the dissolution and melting properties of materials	• perform the melting of the ice cubes experiment<br>• assessment: asked for the reason why the smaller cubes melt faster the pupils should be able to answer that it is due to the higher surface-to-volume ratio	• perform the melting of the ice cubes experiment<br>• perform the rock sugar dissolution experiment; optionally also perform the experiment with the sugar loaf and the the pupils discuss why this experiment fails<br>• assessment: asked for the reason why the smaller cubes melt faster the pupils should be able to answer that it is due to the higher surface-to-volume ratio	• perform the melting of the ice cubes experiment in a simplified way: take 2 ice cubes. One is crashed by a hammer in small pieces. Put the intact ice cube in one glass nd the smashed pieces in another glass. At the end of the lecture the small pieces should have molten to water, whereas the intact ice cube is not fully molten<br>• assessment: asked the question whether the audience understood that the smaller cubes melt faster due to the higher surface-to-volume ratio
Influence of the surface-to-volume ratio on the catalytic properties of materials	• the teacher should either perform the colorimetric assay by him/herself. Alternatively, the pupils may also do the pipetting of the pre-prepared solutions. Especial care has to be taken about wearing googles, gloves, and coated<br>• the reaction equation does not need to be show, it is only important that the pupils see whether a reaction happens or not<br>• assessment: this part is complicated for the pupils, as they have not learned in school what "catalysis" is. Discuss this based on the Supporting Figure S5 and ask by questioning if the general concept is understood	• The pupils should do the pipetting of the pre-prepared solutions. Especial care has to be taken about wearing googles, gloves, and coated<br>• The reaction equation should be discussed<br>• assessment: discuss with the students about the potential use of nanomaterials for catalytic applications. Ask how a catalytic converter in a car could be improved (give as a hint that it depends on the available surface of Pt). Ask why nanomaterials thus could also impose risks. The pupils should be able to tell that the high surface-to-volume ratio may make materials catalytically active and thus reactive	• discuss surface reactivity at the example of a cut avocado: after a while, the surface gets brownish. While a freshly peeled avocado will turn brown only at its outer surface, an avocado cut into small pieces will be fully turn brown<br>• discuss the perspectives for using nanomaterials for applications and the potential risks: mention the catalytic converter of cars, which involves Pt. Pt nanostructured Pt has a higher surface-to-volume ratio and thus performs better. Consequently, to achieve the same performance less nano-particulate Pt is required than bulk Pt and thus less weight. Mention then that also surface which are barely reactive with bulk materials (golden ring), in case of nano-particulate form (gold nanoparticles) they may have measurable surface reactivity (catalytic effects)<br>• assessment: ask for the benefits and risks of nanomaterials. This generally will lead to a discussion about risk-assessment
